# Hypoglycaemia and its associations with diabetes and age-related factors in older home-dwelling people with diabetes

**DOI:** 10.1186/s12877-025-06732-9

**Published:** 2025-12-15

**Authors:** Mari Fløde, Monica Hermann, Jannicke Igland, Ingvild Hernar, Arun K. Sigurdardottir, Eirik Søfteland, Anders Åsberg, Trond Geir Jenssen, Anne Haugstvedt

**Affiliations:** 1 Department of Health and Caring Sciences, Western Norway University of Applied Sciences, P.O. Box 7030, Bergen, N-5020 Norway; 2 Department of Global Public Health and Primary Care, University of Bergen, Bergen, Norway; 3 Department of Internal Medicine, Haukeland University Hospital, Bergen, Norway; 4 School of Health Sciences, University of Akureyri, Akureyri, Iceland; 5 Akureyri Hospital, Akureyri, Iceland; 6 Department of Transplantation Medicine, Oslo University Hospital, Oslo, Norway; 7 Department of Pharmacy, Section for Pharmacology and Pharmaceutical Biosciences, University of Oslo, Oslo, Norway; 8 Institute of Clinical Medicine, University of Oslo, Oslo, Norway

**Keywords:** Diabetes, Older people, Hypoglycaemia, Continuous glucose monitoring, Glycaemic variability, Home care, Polypharmacy

## Abstract

**Background:**

Studies have shown high rates of hypoglycaemia among home-dwelling older people receiving home care services. Hypoglycaemia can lead to severe consequences and in worst case, death. Therefore, knowledge about the factors associated with the occurrence of hypoglycaemia in this patient group is important. This study aimed to investigate the associations between hypoglycaemia and relevant diabetes and age-related factors among individuals with diabetes aged ≥ 65 years who received home care services.

**Methods:**

This prospective observational study included data from blinded continuous glucose monitoring to identify hypoglycaemia and glycaemic variability. Further, data from blood tests (HbA1c, serum creatinine), medication lists, and questionnaires on cognitive function, functional status with need of assistance, and nutritional status were collected. Data analysis was performed using Fisher’s exact tests and unadjusted logistic regression analyses.

**Results:**

In total, 56 individuals participated (52% men, median age 82 years). We identified a statistically significant association between hypoglycaemia and glycaemic variability (*p* < 0.001). All participants with a coefficient of variation (CV) ≥ 36% had undergone at least one hypoglycaemic event during the study period. Also, participants with CV ≥ 27% had higher risk of hypoglycaemia than those with CV < 27% (OR 5.3, 95% CI 1.6, 17.7). Among the 34 participants with HbA1c ≥ 53 mmol/mol, 32% experienced hypoglycaemia. In total, 93% were treated with medications that potentially interfered with the effect of their diabetes medications, and 64% were treated with medications that potentially affected their ability to detect symptoms of hypoglycaemia. The associations between hypoglycaemia and cognitive, functional, or nutritional status is uncertain due to limitations in sample size.

**Conclusions:**

This study highlights a significant association between glycaemic variability and hypoglycaemia among older home-dwelling adults with diabetes receiving home care services. The prevalence of drug-drug interactions raises concerns about diabetes treatment and emphasise the need for targeted interventions to improve safety and care for this vulnerable group of patients.

## Background

Both type 1 and type 2 diabetes pose significant challenges to healthcare systems due to rising prevalences, increasing life expectancies, and associated comorbidities and costs [1]. Research indicates that as much as 20–30% of individuals receiving home care services in Norway have diabetes [2, 3]. In most countries, type 2 diabetes accounts for more than 90% of diabetes cases [4].

Treating and self-managing diabetes in older people is complex, and guidelines emphasise simplifying the medication and avoiding overtreatment [5]. The treatment focuses not only on lowering glucose to satisfactory levels, but also on minimising the risk of hypoglycaemia. In older people, there is often a preference for maintaining slightly higher glucose levels to prevent low levels [5, 6]. Several studies have shown that the occurrence of hypoglycaemia in older people with diabetes is worryingly high [7, 8]. In Norway, our recent study using five-day blinded continuous glucose monitoring (CGM) revealed that one in three older participants who used insulin experienced at least one hypoglycaemic event [8]. Surprisingly, nearly half of the participants who were not on insulin also had at least one hypoglycaemic event [8]. Hypoglycaemia can have severe consequences for older people, including falls, fractures, vascular complications and even death, underscoring the gravity of this issue [7]. Therefore, knowing the risk factors for hypoglycaemia is essential to prevent such serious events.

Several diabetes- and age-related factors influence the risk of hypoglycaemia in older people with diabetes. Both high and low average glucose levels have been shown to be associated with a higher incidence of hypoglycaemia [9, 10]. Also, cognitive decline has been linked to an increased risk of hypoglycaemia [11], and conversely, severe hypoglycaemia has been associated with a greater risk of cognitive decline [12, 13]. Further, missed meals or poor nutrition are shown to be common triggers for hypoglycaemia [12]. In older people with diabetes, polypharmacy is prevalent and strongly related to a higher risk of drug-drug interactions that may affect the risk of hypoglycaemia [13, 14]. For example, the additive pharmacodynamic effects of CNS depressants may induce drowsiness that can mask hypoglycaemic symptoms. Also, the commonly used beta-blockers may increase the risk of hypoglycaemia [15, 16]. Based on this existing knowledge and the high incidence of hypoglycaemic events identified in our previous study among older adults with diabetes receiving home care services [8], we aimed in this study to further investigate the associations between hypoglycaemia and relevant diabetes- and age-related variables.

## Methods

### Study design

This was a prospective observational study with five-days follow-up with continuous glucose monitoring (CGM), among individuals with diabetes aged ≥ 65 years who received home-based healthcare services.

### Setting, study sample and recruitment

We targeted older individuals with diabetes from home care services in a large municipality in Western Norway [8]. Home care services in Norway are legally mandated and publicly funded. These services include health and medical care and are part of primary health care. A specific electronic patient record is used for documenting home care services, which is different from what is used by GPs and in specialist health services. The municipality assesses the extent of care needs by using an instrument that evaluates resources and assistance needs.

Eligibility criteria included age ≥ 65 years, treatment with glucose-lowering medications, ability to provide informed written consent, and ability to communicate in Norwegian. Individuals with diseases that may be underlying causes of glucose instability (such as malfunction of the adrenal cortex, malfunction of the pituitary gland, liver failure, or surgically removed ventricle) were excluded, as well as those who already used CGM and individuals with severe somatic or psychiatric comorbidities (e.g., known end-stage kidney disease, severe heart failure, severe cancer, severe depression or bipolar disorder, psychosis). To identify eligible participants, we obtained information on age, diagnosis of diabetes, and medication from electronic patient records. Study nurses consecutively invited all eligible participants with assumed adequate cognitive function to participate. The evaluation of cognitive function was based on the nurses’ perception of the patient’s cognitive function and their knowledge of the patient over time.

In addition to recruiting participants from the initial list obtained from electronic patient records, the study nurses also continuously enrolled new eligible participants from the lists of new patients in the home care service throughout the recruitment period, from January 2020 to December 2021.

### Data collection

Demographic (sex, living conditions) and clinical data (type of diabetes, age, weight, height, hypoglycaemic events (≤ 3,9 mmol/L) the previous four weeks, medication list and information about the duration of medical treatments) were collected from the electronic patient records.

Medtronic iPro2, a blinded CGM system, and Enlite glucose sensors were used to collect data about the participants’ interstitial fluid glucose levels over five days, with glucose readings tracked every 5 min [17, 18]. The sensor was placed on the participant’s stomach to prevent pressure-induced errors, assuming older people rarely sleep in this position, thereby minimising pressure-related issues. All participants underwent capillary blood glucose measurements three times daily for calibration, and their daily routines and diabetes treatment remained unchanged from usual. In addition, a blood sample was taken during the project period to measure glycaemic control and kidney function. Questionnaires were used to collect data on cognitive function, functional status with the need for assistance, and nutritional status. During the 5-day continuous glucose monitoring (CGM) period, the study nurses completed the questionnaires at the patient`s home.

### Hypoglycaemia outcomes

Hypoglycaemic events were defined as glucose levels ≤ 3.9 mmol/L for at least 15 min and recovery reaching or exceeding 3.9 mmol/L for at least 20 min. Severe hypoglycaemia was defined < 3.0 mmol/L.

### Measures of potential risk factors for hypoglycaemia

Glycaemic variability was described through the coefficient of variation (CV), calculated as the standard deviation of sensor glucose values during the CGM observation period divided by the mean of sensor glucose values in the same period [17]. The established CV cut-off of ≥ 36% was used to explore glycaemic variability [17, 19]. CV < 27% was also evaluated, as values below this threshold have been shown to significantly reduce the risk of hypoglycaemia [19]. Glycaemic control was measured by HbA1c, and kidney function by serum creatinine and estimated glomerular filtration rate (eGFR). The eGFR was determined using the CKD-EPI creatinine Equation (2021) [20].

The database Micromedex was used to identify potential drug-drug interactions based on previous reports on accuracy and comprehensiveness [21, 22]. Drug-drug interactions were classified following the classification system of Micromedex as either contraindicated, major (interaction may be life-threatening or require medical intervention to minimise or prevent adverse effects) or moderate (the interaction may result in exacerbation of the patient’s condition and/or require an alteration in therapy). Only interactions interfering with the effect of the glucose-lowering medication or affecting the ability to perceive symptoms of hypoglycaemia were registered. Interactions registered as “Interfering with the effect of diabetes medication” included both pharmacodynamic and pharmacokinetic drug-drug interactions, resulting in increased or decreased glucose-lowering effect. Interactions registered as “Affecting the ability to perceive symptoms of hypoglycaemia” included both interactions directly affecting the autonomic symptoms of hypoglycaemia (i.e. use of beta blockers in combination with diabetes medication) [15] and pharmacodynamic or pharmacokinetic interactions causing increased CNS depression and thereby affecting awareness and hypoglycaemia sensing. Examples of the latter include pharmacodynamic interactions between, e.g., benzodiazepines and z-hypnotics, or pharmacokinetic interactions causing decreased elimination and thereby increased effect of drugs causing CNS depression, such as, for example, hypnotics.

Cognitive function was measured using the Mini-Mental State Exam (MMSE), a diagnostic tool for cognitive status, impairment, and dementia [23, 24]. It has been translated and validated among Norwegian patients [25]. The MMSE consists of 11 items grouped into six domains: orientation, working memory/registration, concentration/attention, recall, language, and visuospatial. The total score has a maximum of 30 points, where 28–30 indicates normal cognitive function, 25–27 possible cognitive impairment, and ≤ 24 cognitive impairment [25]. MMSE is considered the standard and first-line screening tool for cognitive impairments in older people [26].

Functional status and need for assistance were assessed using a measure from the Norwegian National Register of Municipality Care (*Individbasert pleie- og omsorgsstatistikk - IPLOS*) [27]. The instrument evaluates resources and assistance needs among those who apply for or receive municipal home-based care services and is based on the International Classifications of Functioning, Disability and Health [27]. Seventeen activities of daily living are registered. Each item is scored on a Likert scale from 1 to 5 or found not applicable (score 9). The activities are grouped into five domains with 1–6 questions in each domain: social functioning, cognitive status, maintaining one’s health, household, and self-care. For each domain, a score is calculated as the mean of the scores on each question. After the calculation of domain scores, the score is recoded to indicate the different levels of assistance needed. Values ≤ 2 are recorded as 1.5, indicating some/limited need of assistance. Values >2 and ≤ 3 are recorded as 3, representing a moderate need for assistance. Values >3 are recorded as 4.5, indicating a substantial need for assistance. The mean of the five recoded domain scores is calculated to obtain a total functional level score from 1.5 to 4.5, with higher scores indicating a higher need for assistance [27].

Malnutrition or “at risk of malnutrition” was assessed using the Mini Nutritional Assessment Short Form (MNA-SF) [28]. The MNA-SF evaluates food intake and weight loss over the past three months, current mobility, psychological stress, acute illness, neuropsychological problems such as dementia or depression and body mass index. The scores range from 0 to 14, with a score of ≥ 12 indicating normal nutritional status and ≤ 11 suggesting possible malnutrition [28, 29].

### Data analysis

Demographic and clinical characteristics were described for the total sample and separately for participants who experienced one or more hypoglycaemic events during the five-day study period and those who did not.

For the subgroup of participants with at least one hypoglycaemic event, we detailed the number of events, the number of events in different glucose intervals, the total duration of hypoglycaemia, glucose variability (both continuous and categorical), HbA1c (mmol/mol [%]), eGFR (mL/min/1.73 m²), and BMI (kg/m²). Due to the small sample size and skewed data, the median (range) was reported for continuous variables. Glucose variability was reported using the CV and range (min-max). To visualise glucose variability, we created a strip plot showing distribution of CV-values among participants with and without hypoglycaemic events. The strip plot displayed the coefficient of variation for glucose measurements as jittered points to avoid overlap, with mean CV and confidence intervals displayed. Each dot represents the CV value for a single patient. The mean CV was compared between the two groups using an independent sample t-test.

Bivariate logistic regression analyses were conducted to examine the associations of the occurrence of hypoglycaemia with HbA1c, glycaemic variability, cognitive function, functional status with need of assistance, nutritional status, and kidney function. Results are reported as odds ratios (OR) and 95% confidence intervals (CI). No adjustments were made because of the small sample size.

Statistical evaluation of differences in medication use and prevalence of drug-drug interactions between participants with and without hypoglycaemia was done by Fisher`s exact test. We used STATA/MP 18.0 for the statistical analyses.

## Results

### Sample characteristics

Demographic, functional and clinical characteristics of the 56 participants are shown in Table 1. In short, the median age was 82 (range 65–99) and consisted of 52% men. In total, 64% were treated with insulin, the majority in combination with oral glucose-lowering medications. The remaining 36% used various combinations of oral glucose-lowering medications (Table 1). Among the 21 participants experiencing hypoglycaemia, a total of 37 events were recorded, with each participant having between 1 and 5 events. Seventeen events (46%) were nocturnal, 16 events (76%) were level 1 hypoglycaemia (3.0–3.8 mmol/L), and 5 (24%) were level 2 hypoglycaemia (< 3.0 mmol/L). The level 1 events lasted from 15 min to 8 h and 17 min (median 1 h and 15 min), while the level 2 events lasted from 30 min to 6 h and 25 min (median 1 h and 30 min). Thirty-five participants did not experience hypoglycaemic events during the study period (Table 1). Among those who did not experience hypoglycaemia during the five days on CGM, three had experienced one event with recorded blood glucose level ≤ 3.9 mmol/L documented in the electronic record during the four weeks prior to the data collection. None of these events were classified as severe.

**Table 1:** Clinical and functional characteristics of the population

Variables	Total sample *N* = 56	Hypoglycaemia *n* = 21	No hypoglycaemia *n* = 35
Age (years), median (min-max)	82 (65–99)	83 (67–94)	81 (65–99)
Sex, *n* (%)			
Male	29 (52)	10 (48)	19 (54)
Female	27 (48)	11 (52)	16 (46)
Living alone, *n* (%)	40 (71)	18 (86)	23 (66)
Type of diabetes, *n* (%)			
Type 1	7 (12)	2 (10)	5 (14)
Type 2	49 (88)	19 (90)	30 (86)
Diabetes duration (years), median (min-max)	16 (1–49)	16 (1–39)	15 (1–49)
Type of treatment			
Only insulin, *n* (%)	13 (23)	6 (29)	7 (20)
Type 1 diabetes	5 (9)	2 (10)	3 (9)
Type 2 diabetes	8 (14)	4 (19)	4 (11)
Insulin combined with other glucose-lowering medication, *n* (%)	23 (41)	6 (29)	17 (49)
Glucose-lowering medication without insulin, *n* (%)^a^	20 (36)	9 (42)	11 (31)
eGFR (mL/min/1.73 m²), median (min-max) ^b^	69 (9–123)	61 (22–110)	80 (9–123)
Body mass index (kg/m^2^), median (min-max)	27 (19–41)	24 (21–39)	28 (19–41)
Glycaemic variability (CV%), median (min-max)^c^	25 (14–39)	29 (14–39)	23 (14–34)
HbA1c (mmol/mol), median (min-max) ^d^	57 (34–108)	52 (38–80)	60 (34–108)
HbA1c (%), median (min-max)	7.4 (5.3–12.0)	6.9 (5.6–9.5)	7.6 (5.3–12.0)
Cognitive function (MMSE, 0–30), median (min-max) ^**e**^	25 (12–30)	24 (18–30)	25 (12–30)
Nutritional status (MNA, 0–14), median (min-max) ^f^	13 (6–14)	13 (7–14)	13 (6–14)
Need for assistance (IPLOS, 1–5), median (min-max) ^g^	2.4 (1.5–4.2)	2.1 (1.5–4.2)	2.4 (1.5–3.9)
Total number of medications, median (min-max)	10 (5–24)	9 (5–24)	10 (6–21)

Missing data: HbA1c, *n*=6 and eGFR, *n*=7; body mass index, *n*=3

^a^Glucose-lowering medications other than insulin: metformin, *DPP-4* dipeptidyl peptidase IV, inhibitors, *GLP-1* glucagon-like peptide-1, receptor agonists, *SGLT2* sodium-glucose transport protein 2 and/or inhibitors, sulphonylureas (*n*=2)

^b^eGFR, the *eGFR* estimated glomerular filtration rate

^c^Glycaemic variability is measured using the *CV* coefficient of variation

^d^HbA1c,Glycated Haemoglobin

^e^*MMSE* Mini-Mental State Examination. The total score 0–30; 28–30 indicates normal cognitive function, 25–27 possible cognitive impairment, and ≤24 cognitive impairment

^f^*MNA-SF* Mini Nutritional Assessment Short Form. The total 0–14, ≥12 indicates normal nutritional status and ≤11 possible malnutrition

^g^IPLOS, Norwegian National Register of Municipality Care (Individbasert pleie- og omsorgsstatistikk). Total functional level score 1.5–4.5.5.5, with higher scores indicating a greater need for assistance

### Disease-related variables and hypoglycaemia

**Fig. 1:**
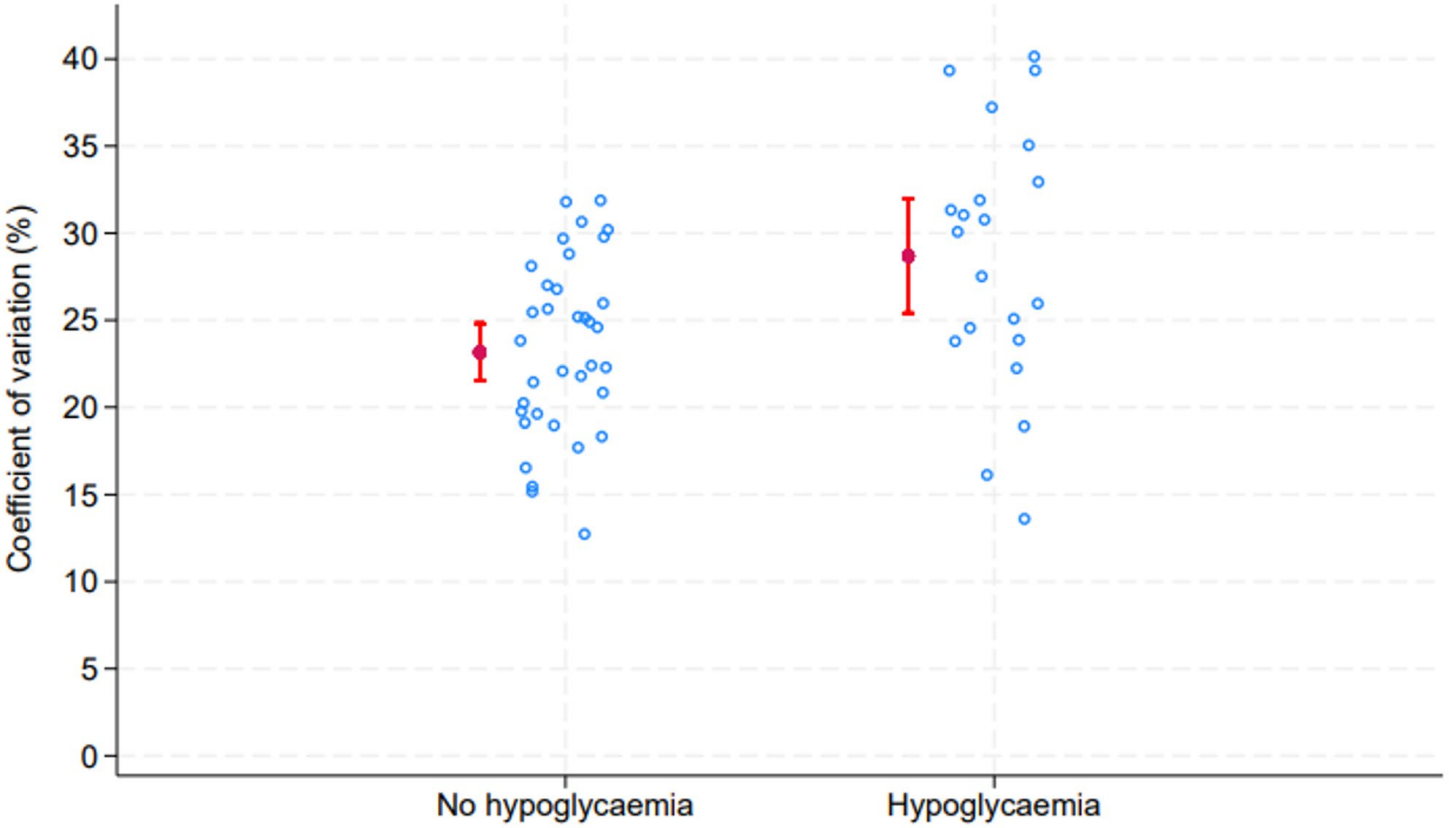
Strip-plot indicating glucose variability measured in terms of coefficient of variation. Red markers with whiskers indicate means and 95% confidence intervals among those with hypoglycaemic events and those without hypoglycaemic events (*p*-value for difference in means *p* = 0.001). No hypoglycaemia, *n*=35, Hypoglycaemia, *n*=21

The mean CV for glucose variability was significantly higher among participants with hypoglycaemia compared to participants without hypoglycaemia (*p* < 0.001) (Fig. 1). All participants with a CV ≥ 36% had undergone at least one hypoglycaemic event during the study period (Table 2). Also, those with CV ≥ 27% had higher odds for one or more hypoglycaemia events than those with CV < 27% (OR 5.3, 95% CI 1.6, 17.7).

**Table 2:** Diabetes- and age-related factors in the participants with hypoglycaemia compared to those without hypoglycaemia during the study period

Variables	Total *N* = 56	Hypoglycaemia *n* = 21	No hypoglycaemia *n* = 35	Odds Ratio for hypoglycaemia (95% CI)
Glycaemic variability (CV) ^a^				
CV < 36%, *n* (%)	52 (100)	17 (33)	35 (67)	Ref.
CV ≥ 36%, *n* (%)	4 (100)	4 (100)	0 (0)	NE^g^
CV < 27%, *n* (%)	37 (100)	9 (24)	28 (76)	Ref.
CV ≥ 27%, *n* (%)	19 (100)	12 (63)	7 (37)	5.3 (1.6, 17.7)
HbA1c ^b^				
HbA1c < 53 mmol/mol, *n* (%)	22 (100)	10 (45)	12 (55)	1.7 (0.6, 5.3)
HbA1c ≥ 53 mmol/mol, *n* (%)	34 (100)	11 (32)	23 (68)	Ref.
HbA1c < 64 mmol/mol, *n* (%)	33 (100)	13 (39)	20 (61)	1.2 (0.4, 3.7)
HbA1c ≥ 64 mmol/mol, *n* (%)	23 (100)	8 (35)	15 (65)	Ref.
Cognitive function (MMSE, 0–30)^c^				
No cognitive impairment (> 24), *n* (%)	30 (100)	8 (27)	22 (73)	Ref.
Cognitive impairment (≤ 24), *n* (%)	26 (100)	13 (50)	13 (50)	2.7 (0.9, 8.4)
Nutritional status (MNA, 0–14)^d^				
Normal nutritional status (≥ 12), *n* (%)	44 (100)	17 (39)	27 (61)	Ref.
At risk or malnourished (≤ 11), *n* (%)	12 (100)	4 (33)	8 (67)	0.8 (0.2, 3.0)
Functional status and need of assistance (IPLOS, 1–5)^e^				
Some/limited need for assistance (< 3.0), *n* (%)	44 (100)	16 (36)	28 (64)	Ref.
Moderate/substantial need of assistance (≥ 3.0), *n* (%)	12 (100)	5 (42)	7 (58)	1.3 (0.3, 4.6)
Kidney status ^f^				
eGFR ≥ 60 mL/min/1.73 m², *n* (%)	30 (100)	9 (30)	21 (70)	Ref.
eGFR < 60 mL/min/1.73 m², *n* (%)	19 (100)	9 (47)	10 (53)	2.1 (0.6, 6.9)

^a^Glycaemic variation is measured using the *CV* coefficient of variation

^b^HbA1c, Glycated Haemoglobin

^c^*MMSE* Mini-Mental State Examination

^d^*MNA-SF* Mini Nutritional Assessment Short Form

^e^IPLOS: Norwegian National Register of Municipality Care (Individbasert pleie- og omsorgsstatistikk)

^f^Kidney status: the *eGFR* estimated glomerular filtration rate *Missing data: HbA1c, *n* = 6, MMSE, *n* = 2, Kidney status. *n* = 7 eGFR = 7

^g^*NE* not estimable

Among the 22 participants with HbA1c < 53 mmol/mol (7.0%), 45% experienced hypoglycaemia during the study period (Table 2). Among the 34 participants with HbA1c ≥ 53 mmol/mol (7.0%), 32% experienced hypoglycaemia. The association between hypoglycaemia and HbA1c was inconclusive with wide confidence intervals (OR 1.7, 95% CI 0.6, 5.3) (Table 2). The median number of medications prescribed to the participants was 10 (range 5–24) (Table 3). In total, 76% of those who experienced hypoglycaemia were treated with psychotropic medications compared to 51% among those who did not experience hypoglycaemia. Furthermore, potential drug-drug interactions interfering with the effect of the diabetes medication were identified in 93% of the participants. Additionally, 64% of the participants were exposed to one or more drug-drug interactions that may have impaired their ability to recognize symptoms of hypoglycaemia, with 46% of these individuals using beta-blockers (Table 3).

**Table 3:** Use of medication and drug-drug interactions

	Total sample (*N* = 56)	Hypoglycaemia (*n* = 21)	No hypoglycaemia (*n* = 35)
Medication use			
Total number of medications, median (min-max)	10 (5–24)	9 (5–24)	10 (6–21)
Number of diabetes medications, median (min-max)	2 (1–5)	2 (1–5)	2 (1–5)
Number of psychotropic medications, median (min-max)	1 (0–6)	1 (0–3)	1 (0–6)
Patients using ≥ 1 psychotropic medication, *n* (%)	34 (61)	16 (76)	18 (51)
Patients using beta blockers, *n* (%)	26 (46)	9 (43)	17 (51)
Drug-drug interactions			
Number of drug-drug interactions per individual, median (min-max)			
*Total number of interactions*	6 (0–25)	6 (0–25)	7 (2–22)
*Moderate interactions*	3 (0–10)	2 (0–9)	4 (1–10)
*Major interactions*	3 (0–16)	3 (0–16)	2 (0–14)
*Contraindications*	0 (0–2)	0 (0–1)	0 (0–2)
Individuals with ≥ 1 interaction interfering with the effect of diabetes medication, *n* (%)	52 (93)	19 (90)	33 (94)
*Interactions between different diabetes medications*	25 (45)	12 (57)	13 (37)
*Interactions between diabetes medication and other medication*	51 (91)	18 (86)	33 (94)
Individuals with ≥ 1 interaction affecting the ability to perceive symptoms of hypoglycaemia, *n* (%)	36 (64)	13 (62)	23 (66)

Data are median (min-max) and *n* (%)

### Age-related variables and hypoglycaemia

In total, 26 (46%) of the participants had cognitive impairment (MMSE ≤ 24) and 50% of them had hypoglycaemic events during the study period compared to 33% among those without cognitive impairment. However, the association between hypoglycaemia and cognitive impairment was uncertain due to limitation in sample size (OR 2.7, 95% CI 0.9, 8.4) in this study.

Regarding functional status and need of assistance, the total score was 2.4 (min-max 1.5–4.2) (Table 1), and 12 (21%) participants reported scores indicating moderate/substantial need of assistance (≥ 3.0) related to the measured domains (Table 2). The remaining 44 participants required limited/some need of assistance (< 3.0). The most common activities in which our sample reported scores indicating declined function and moderate/substantial need for assistance were “maintaining one`s health” (*n* = 50), “self-care” (*n* = 23), and “household” (*n* = 38) (Table 4). However, we did not identify a statistically significant association between hypoglycaemia and these scores (OR 1.3, 95% CI 0.3, 4.6) (Table 2). Among the 12 participants at risk or malnourished (MNA-SF ≤ 11.0) and those 44 with normal nutritional status, the number with hypoglycaemic events was 4 and 17, respectively (OR 0.8, 95% CI 0.2, 3.0) (Table 2).

**Table 4:** Level of assistance needs due to functional decline

Level of assistance needs in the domains	Total *N* = 56	Hypoglycaemia *n* = 21	No hypoglycaemia *n* = 35	Odds Ratio for hypoglycaemia (95% CI)
Cognitive status, *n* (%)				
Some/limited need of assistance	45 (100)	17 (38)	28 (62)	Ref.
Moderate or substantial need of assistance	11 (100)	4 (36)	7 (64)	0.9 (0.2, 3.7)
Maintaining one’s health, *n* (%)				
Some/limited need of assistance	6 (100)	2 (33)	4 (67)	Ref.
Moderate or substantial need of assistance	50 (100)	19 (38)	31 (62)	1.2 (0.2, 7.4)
Self-care, *n* (%)				
Some/limited need of assistance	33 (100)	15 (45)	18 (55)	Ref.
Moderate or substantial need of assistance	23 (100)	6 (26)	17 (74)	0.4 (0.1, 1.4)
Social functioning, *n* (%)				
Some/limited need of assistance	48 (100)	18 (38)	30 (62)	Ref.
Moderate or substantial need of assistance	8 (100)	3 (38)	5 (62)	1.0 (0.2, 4.7)
Household, *n* (%)				
Some/limited need of assistance	18 (100)	7 (39)	11 (61)	Ref.
Moderate or substantial need of assistance	38 (100)	14 (37)	24 (63)	0.9 (0.3, 2.9)

Some/limited need for assistance: score < 3.0, Moderate or substantial need of assistance: score ≥ 3,0

Among the participants experiencing hypoglycaemic events, 47% had mild to severely decreased kidney function (eGFR, < 60 mL/min/1.73 m²) (Table 2). In this group, four had moderate to severe decreased kidney function (eGFR ≤ 44 mL/min/1.73m^2^) and two had severely decreased kidney function (eGFR < 30mL/min/1.73m^2^).

## Discussion

Although several studies have looked at the risk of hypoglycaemia in older people [7], to our knowledge, this is the first study primarily focusing on home-dwelling older people receiving home care services. We identified a statistically significant association between hypoglycaemia and glycaemic variability, with a higher incidence of hypoglycaemia observed among participants exhibiting substantial glycaemic variability. Associations with HbA1c and cognitive impairment were inconclusive due to lack of statistical power, but the observed frequency of hypoglycaemia was higher among persons with lower HbA1c and among persons with cognitive impairment. All participants were subjected to polypharmacy, and more than 90% of the participants were treated with medications that potentially interfered with either their ability to detect hypoglycaemic symptoms or the efficiency of their diabetes medications. Also, half of the participants with hypoglycaemia had impaired kidney function.

Regarding glycaemic variability, all participants with CV ≥ 36% experienced hypoglycaemic events. This corroborates with other studies, showing that the 36% CV threshold is a reliable risk factor of hypoglycaemic events across age groups [17, 30, 31]. Compared to younger people, it has been shown that older individuals with diabetes and high glucose variability are at a greater risk of hypoglycaemia [32]. Furthermore, a review from 2023 showed that CV below 27% significantly reduced the risk of hypoglycaemia [19]. In our sample, 76% of individuals with a CV < 27% did not experience any hypoglycaemic event during the study period. Given that the participants in this study had a median age of 82 years, with the oldest participant being 99, our findings confirm glucose variability as a significant risk factor for hypoglycaemia in older adults.

National and international guidelines provide limited recommendations on managing glucose regulation in older people with diabetes, often only suggesting a slightly higher HbA1c to prevent hypoglycaemia [5, 33]. However, we identified a fairly high incidence of hypoglycaemia not only among those with HbA1c < 53 mmol/mol (< 7.0%), but also among those with HbA1c ≥ 53 mmol/mol (≥ 7.0%) and those with HbA1c ≥ 64 mmol/mol (≥ 8.0%). This highlights the limitation of relying solely on HbA1c to adjust glucose-lowering treatments in older people to prevent hypoglycaemia, i.e., allowing for a higher HbA1c does not necessarily prevent hypoglycaemia [34, 35].

This study indicates that CGM may be beneficial for older individuals with diabetes. Traditional finger-prick blood glucose monitoring (BGM) provides only a snapshot of the glucose level and falls short of accurately detecting and recording glycaemic variability and hypoglycaemia. CGM has been shown to detect more than 90% of hypoglycaemic events not detected with BGM [36]. Additionally, the association between CV and hypoglycaemia indicates a need to monitor glucose fluctuations more effectively than is currently done with BGM [37, 38]. Furthermore, CGM can significantly improve glycaemic control by reducing hypoglycaemia, particularly at night, which is especially beneficial for individuals aged 65 and older [34]. Although several studies have shown that CGM data for 5–14 days provides reasonable estimates of glucose metrics for three months [39–41], municipal healthcare services rarely utilise CGM. They often lack the necessary knowledge and access [42], possibly due to both structural and economic limitations.

In our study, all participants used five or more medications, while 50% used 10 or more medications, often classified as “excessive polypharmacy” [43]. The prevalence of polypharmacy is higher in our study compared to previous reports in older home-dwelling people with diabetes [13, 44]. Still, it was not surprising, as persons receiving home care services often have multiple chronic conditions requiring medical treatment. Also, the prevalence of potential drug-drug interactions was high, with more than 90% having one or more moderate or major potential drug-drug interactions interfering with the effect of diabetes medication. However, these potential interactions may have been adjusted for by dose reductions, of which we have no information. A previous large study from Scotland utilising health care records showed that the use of several classes of psychotropic drugs increased the risk of adverse health outcomes, among them hospitalisations for hypoglycaemia in individuals with type 1 diabetes [45]. In our study, we also observed a trend towards a higher number of individuals using psychotropic drugs in the group that experienced hypoglycaemia. In summary, the number of medications, use of psychotropic drugs and the high prevalence of potential drug-drug interactions call for increased attention to optimising medication use in this group of patients.

We consider it concerning that 62% of the participants with hypoglycaemic events had cognitive impairment according to the MMSE measurement. It has also previously been shown that those with cognitive impairment have a threefold higher incidence of severe hypoglycaemic events [46]. Conversely, it has also been shown that severe hypoglycaemia can lead to a decrease in cognitive function [47], indicating a potentially vicious circle. Hence, it is important to avoid hypoglycaemia in older individuals with cognitive impairment.

The widely reported link between impaired kidney function and hypoglycaemia [7, 48, 49] is also demonstrated in our study, as 43% of the participants with hypoglycaemia had impaired kidney function (eGFR < 60 mL/min/1.73 m^2^). Although we aimed to exclude individuals with severe kidney failure during the recruitment period, the study nurses included some individuals with a previously unidentified significantly reduced kidney function. Even a mild reduction in kidney function is shown to be associated with increased incidence and severity of hypoglycaemia [50, 51]. None of the participants with impaired kidney function used metformin or sodium-glucose transport protein 2 (SGLT2) inhibitors that are contraindicated according to guidelines [5].

### Strengths and limitations

A strength of the study is the collection of blinded CGM data, ideal for identifying hypoglycaemia and measuring CV. The approach enables comprehensive retrospective analysis by identifying patterns without interference from immediate feedback. The MNA questionnaire was completed by the nurse at the patient’s home. Only part 1 of the MNA questionnaire was used, eliminating the risk of recall bias. The study’s primary limitation is the relatively small sample size, which likely affected our ability to detect significant differences between groups. There is a possibility that there are associations between the studied variables that we did not detect. Challenges with access to CGM sensors (phased out products) prevented us from extending the study period beyond December 30, 2021. The COVID-19 regulations also restricted the recruitment of participants to the study. Incomplete health records may have introduced uncertainty about the diagnosis of diabetes type. Although generalisation to other settings and populations should be done with caution, the study provides valuable insights into a diabetes population of older people receiving home care services, which is a research field with a limited knowledge base.

## Conclusion

This study highlights a significant association between glycaemic variability and hypoglycaemic events among older home-dwelling adults with diabetes receiving home care services. Hypoglycaemia was not only prominent in those with lower HbA1c values, but also in those with higher HbA1c. The prevalence of potential drug-drug interactions raises concerns about diabetes treatment and emphasise the need for targeted interventions to improve safety and care for this vulnerable group of patients.

## Data Availability

Data are available from the corresponding author upon reasonable request.
